# ICIRD: Information-Principled Deep Clustering for Invariant, Redundancy-Reduced and Discriminative Cluster Distributions

**DOI:** 10.3390/e27121200

**Published:** 2025-11-26

**Authors:** Aiyu Zheng, Robert M. X. Wu, Yupeng Wang, Yanting He

**Affiliations:** 1 School of Electronic Information and Engineering, Taiyuan University of Science and Technology, Taiyuan 030024, China; yupengwang@tyust.edu.cn (Y.W.); yantinghe@tyust.edu.cn (Y.H.); 2 School of Computer Science and Technology, Taiyuan University of Science and Technology, Taiyuan 030024, China; 3 Faculty of Engineering and Information Technology, University of Technology Sydney, Sydney 2007, Australia; mingxuan.wu@uts.edu.au

**Keywords:** deep clustering, contrastive learning, discriminative learning, information-principled deep clustering, discriminative distribution sharpness, multi-view inter-cluster distribution redundancy reduction

## Abstract

Deep clustering aims to discover meaningful data groups by jointly learning representations and cluster probability distributions. Yet existing methods rarely consider the underlying information characteristics of these distributions, causing ambiguity and redundancy in cluster assignments, particularly when different augmented views are used. To address this issue, this paper proposes a novel information-principled deep clustering framework for learning invariant, redundancy-reduced, and discriminative cluster probability distributions, termed ICIRD. Specifically, ICIRD is built upon three complementary modules for cluster probability distributions: (i) conditional entropy minimization, which increases assignment certainty and discriminability; (ii) inter-cluster mutual information minimization, which reduces redundancy among cluster distributions and sharpens separability; and (iii) cross-view mutual information maximization, which enforces semantic consistency across augmented views. Additionally, a contrastive representation mechanism is incorporated to provide stable and reliable feature inputs for the cluster probability distributions. Together, these components enable ICIRD to jointly optimize both representations and cluster probability distributions in an information-regularized manner. Extensive experiments on five image benchmark datasets demonstrate that ICIRD outperforms most existing deep clustering methods, particularly on fine-grained datasets such as CIFAR-100 and ImageNet-Dogs.

## 1. Introduction

Deep clustering refers to a family of methods that jointly learn feature representations and cluster assignments through neural networks to produce semantically meaningful and discriminative clusters [1]. This paradigm has shown broad potential in visual, graph, and biological data, where clustering enables structural pattern discovery and semantic organization [2,3]. In numerous advanced deep clustering algorithms, the cluster probability distribution plays a central role as the functional carrier of the clustering objective [4]. Its differentiable probabilistic form characterizes an interpretable and measurable correspondence between samples and clusters, while in neural network models, it is generated from learned feature representations, thereby building a relational bridge between representation learning and clustering optimization. With the incorporation of data augmentation and self-supervised learning into deep clustering frameworks, the performance of deep clustering has achieved unprecedented improvement [5].

However, existing methods still fail to sufficiently model the information characteristics of cluster probability distributions, especially under cross-view scenarios. Consequently, during practical training, these distributions are often disturbed by representation perturbations, augmentation discrepancies, and inter-class redundancy, leading to uncertain predictions, insufficient separability, and inconsistency across augmentations. This phenomenon is evident in representative early methods: DEC [6] iteratively refines embeddings and cluster assignments via a self-training target distribution, which can propagate pseudo-label errors and is sensitive to clustering hyperparameters; JULE [7] couples agglomerative clustering with a CNN in a recurrent loop and is vulnerable to noise and early merge decisions; DeepCluster [8] uses offline k-means to generate hard pseudo-labels for end-to-end training, but assignments can be unstable across iterations and sensitive to augmentation choices; IIC [9] maximizes mutual information of paired augmentations to enforce consistency, but performance hinges on augmentation design and captured correlations. Accordingly, stabilizing the modeling of the cluster probability distribution is a key challenge for improving robustness and performance in deep clustering.

Building on the advances of contrastive learning [10,11,12], recent works have aimed to alleviate the aforementioned limitations by strengthening the discriminability and consistency of cross-view cluster distributions in joint representation–clustering frameworks. Contrastive Clustering (CC) [13] employs instance-level and cluster-level contrast to reduce augmentation-induced prediction bias. DCRN [14] reduces redundancy by minimizing dual correlations at the feature and cluster levels. DeepCluE [15] integrates multi-layer ensemble clustering with contrastive representations to enhance clustering robustness and accuracy. DHCL [16] enforces hierarchical contrastive constraints across global and local views, enhancing both intra-cluster compactness and cross-view consistency. CCGCC [17] further models the information characteristics of cluster distributions through cross-cluster and graph-level contrastive objectives.

Nevertheless, most recent methods constrain the cluster probability distribution from only a single or partial perspective, lacking a systematic and comprehensive modeling paradigm. On the other hand, approaches based on cross-view cluster distribution contrast often overlook the cluster alignment problem. When the cluster assignments of the same instance are inconsistent across different views, such contrastive operations may instead undermine the stability and accuracy of the model [5]. Therefore, there remains considerable room for exploration in the field of deep clustering.

To address the aforementioned challenges, this paper proposes an information-principled deep clustering framework for learning invariant, redundancy-reduced, and discriminative cluster probability distributions, termed ICIRD. Specifically, ICIRD models clustering as maximizing discriminative mutual information under a constrained information channel, thereby achieving a balance between information preservation and redundancy suppression. First, conditional entropy is minimized to enhance the certainty and discriminability of cluster assignments. Second, inter-cluster mutual information is minimized within each view to suppress statistical redundancy and further improve cluster separability. Finally, cross-view mutual information is maximized to reinforce the semantic invariance and stability of cluster assignments. In addition, a contrastive representation mechanism with both discriminative and information-bottleneck properties is introduced to provide stable and reliable feature inputs for the cluster probability distributions. ICIRD realizes a unified information-principled learning paradigm that bridges representation and clustering learning, enabling the model to learn sharp, independent, and consistent cluster distributions, thereby improving the stability and robustness of the clustering results. The major contributions of this work are summarized as follows:

- A unified information-principled deep clustering framework, termed ICIRD, is proposed. It systematically imposes information constraints on the cluster probability distributions under data augmentation scenarios, covering discriminability, redundancy reduction, and cross-view invariance.
- Three complementary information-principled modules are designed for the cluster probability distribution. The DDS (Section 4.2) module minimizes conditional entropy to enhance assignment certainty and sharpness. The MIDR (Section 4.3) module suppresses redundancy of cluster distributions by minimizing inter-cluster mutual information within each view. The CIDC (Section 4.4) module maximizes cross-view mutual information to preserve the semantic structural stability of cluster assignments.
- Extensive experiments conducted on multiple benchmark datasets demonstrate that ICIRD achieves superior clustering performance compared with state-of-the-art methods. In addition, an analysis of cross-view IDR further shows that the view alignment strategy can partially alleviate the structural distortion caused by cross-view IDR.

The remainder of this paper is structured as follows. Section 2 reviews related studies about ICIRD. Section 3 introduces the preliminaries, including the fundamental information-theoretic concepts and problem definitions of ICIRD. Section 4 presents the proposed ICIRD framework and its information-principled objective formulation. Section 5 provides comprehensive experimental validation of ICIRD across multiple benchmarks. Section 6 illustrates the applications and scalability of ICIRD. Section 7 summarizes the main contributions and conclusions of this work.

## 2. Related Works

### 2.1. Deep Clustering

The core idea of deep clustering is to use deep neural networks to learn clustering-oriented representations and jointly optimize them with clustering objectives, overcoming the limitations of traditional methods on high-dimensional, nonlinear data [1,5]. Early approaches fall into three paradigms: embedding-based, generative-based and pseudo-labeling-based schemes [2,3]. DEC [6] first integrated deep embeddings with self-training to iteratively refine soft assignments. DAC [18] reformulated clustering as pairwise classification with adaptive pseudo-labels. DeepCluster [8] alternated between *k*-means pseudo-labeling and model updating for large-scale training.

These methods still face two core limitations: (i) Alternating optimization often introduces mismatches between feature and cluster spaces, where pseudo-label noise causes semantic drift and fuzzy boundaries. (ii) The absence of explicit modeling at the cluster-probability level limits the controllability of soft assignments. To overcome these issues, IIC [9] maximizes mutual information between augmented views of the same instance to prevent collapse and enforce probability-level consistency. ProtoCon [19] applies prototypical consistency and online pseudo-label refinement to mitigate noise and stabilize decision boundaries. Despite these advances, a unified theoretical framework that jointly ensures sharpness, independence, and cross-view consistency of probabilistic predictions remains elusive.

### 2.2. Mutual Information for Deep Clustering

Mutual Information (MI) measures the shared information between random variables and serves as a key objective in unsupervised and self-supervised learning. In deep models, direct MI computation is intractable, so it is typically approximated by differentiable bounds optimized with neural discriminators or contrastive objectives. Representative methods include NWJ [20], MINE [21] and InfoNCE [10]. Besides, Deep InfoMax [22] extends MI by maximizing local–global MI to learn discriminative representations. Together, these approaches establish a unified framework that transforms MI into trainable objectives for representation learning and clustering.

In clustering, Mutual Information (MI) serves as a natural and interpretable optimization target. IMSAT [23] maximizes MI between inputs and discrete outputs with augmentation consistency; DCCM [24] extends MI to triplet interactions for better cluster discrimination; and DDC [25] maximizes MI between inputs and cluster labels with a semantic generator for interpretable clustering. MIMC [26] generalizes MI to multi-view settings, jointly optimizing completeness, compactness, and diversity while disentangling common and private information. DIB [27] estimates mutual information through kernel Gram matrices to realize compact and consistent representations for multi-view clustering. SDCIB [28] integrates contrastive and information-bottleneck objectives across feature and cluster levels to enhance accuracy and robustness in multi-modal clustering.

Overall, MI in deep clustering has progressed from representation-level to distribution-level modeling and from single-view to cross-view formulations, aligning with the separability and consistency of soft assignments. Yet a smooth transition from self-supervised to clustering-oriented representations remains unrealized, motivating the introduction of clustering-oriented information alignment for joint optimization between the encoder and clustering head—the central focus of this study.

### 2.3. Deep Contrastive Clustering

Contrastive deep clustering extends contrastive learning by constructing positive and negative pairs through data augmentations or multi-view generation, learning discriminative and cluster-friendly representations without labels [11,12]. Core designs include the following: (i) combining instance-level and cluster-level contrast to constrain both sample and assignment spaces, and (ii) maximizing cross-view mutual information to ensure distributional consistency under augmentations. These mechanisms are often integrated with InfoNCE loss, memory queues, and multi-view augmentation for scalable and stable training [12,29,30], while debiasing strategies further mitigate negative-sampling and long-tail effects [31].

Methodologically, CC [13] applies contrastive objectives at both instance and cluster levels and introduces column-space contrast on the assignment matrix to mitigate semantic drift and representation collapse. Prototype-based approaches further stabilize learning: SwAV [29] replaces explicit negatives with swapped assignments for robust large-scale pretraining, and PCL [30] jointly discovers prototypes and performs instance contrast to enhance semantic aggregation. To refine pseudo-labels, SCAN [32] enforces neighbor consistency, SPICE [33] filters labels via semantic confidence, and ProtoCon [19] integrates prototype consistency for online clustering. For structured data, GCC combines graph contrast with cluster-level optimization [34], DCRN reduces sample-level and feature-level redundancy [14]. SACC [35] aligns samples across augmentations using adaptive cluster-level contrast to enhance semantic consistency, while IcicleGCN [36] incorporates graph convolutional reasoning into instance–cluster contrast to exploit relational dependencies in structured data. CoHiClust [37] learns hierarchical cluster structures through contrastive representation learning and a differentiable tree-based head.

While contrastive deep clustering effectively improves representation discriminability and assignment consistency by coupling instance-level and cluster-level objectives, two common gaps persist: (i) Constraints in the feature domain and in the probability-output (clustering-head) domain are often optimized in a loosely coupled manner, lacking a unified treatment of cluster independenceand distributional stability. (ii) Pseudo-label noise and strong augmentations can amplify instability in closed-loop training, impeding early-stage convergence. Motivated by these issues, this paper proposes an information-principled framework that directly enforces sharpness, inter-cluster independence, and cross-augmentation consistency at the clustering head, while leveraging contrastive learning to stabilize the representation space, thereby achieving coordinated optimization across the probability and feature domains.

## 3. Preliminaries

### 3.1. Information-Theoretic Preliminaries

Let the input random variable *X* be transformed via a parameterized mapping into an intermediate representation *Z*, which is then projected through a decision mapping to yield the output random variable *Y*. This setup induces a two-layer Markov chain:

(1) X→Z→Y.

According to the *Data Processing Inequality* (DPI) [38], the flow of information satisfies:

(2) I(X;Y)≤I(X;Z),

indicating that the discriminative information contained in the output cannot exceed that of the learned representation.

In the context of discriminative clustering [4], *Y* is interpreted as a latent random variable representing the cluster assignment of each sample, and its distribution reflects the underlying cluster structure. The natural measure of discriminative dependence between *X* and *Y* is the mutual information, which can be expressed as:

(3) I(X;Y)=H(Y)−H(Y∣X),

where H(Y) denotes the entropy of the marginal cluster distributions, and H(Y∣X) reflects the conditional entropy associated with each prediction.

The two-layer Markov chain suggests that the representation *Z* should retain the information in *Z* that is most relevant for predicting *Y*, while filtering out irrelevant variability. In parallel, a discriminative perspective emphasizes maximizing I(X;Y) appropriately balancing H(Y) and H(Y∣X). By jointly considering the discriminative clustering and the Information Bottleneck (IB) principle [39], ICIRD preserves relevant information while reducing redundancy, yielding cluster predictions that are both confident and robust [22].

### 3.2. Problem Definition

The problem addressed by ICIRD is defined as follows: given an unlabeled dataset $X={\left\{x_{i}\right\}}_{i=1}^{N}$ with a predefined number of clusters *K*, the model consists of a parameterized encoder $f_{\theta }:X\;\to \;R^{d}$, a clustering head $g_{\phi }:R^{d}\;\to \;\Delta^{K-1}$ (where $\Delta^{K-1}$ denotes the *K*-class probability simplex), and a contrastive head $c_{\rho }:R^{d}\;\to \;R^{m}$ for representation-level contrastive learning. Denote the conditional probability output of the model for *X* is $P_{\theta ,\phi }\left(Y|X\right)=g_{\phi }\left(f_{\theta }\left(X\right)\right)$, where $p_{\theta ,\phi }\left(y_{k}|x_{i}\right)={\left[g_{\phi }\left(f_{\theta }\left(x_{i}\right)\right)\right]}_{k}$ and the predicted cluster assignment is $\hat{Y}=\arg\max_{k}P_{\theta ,\phi }\left(Y|X\right)$, where k∈K. Under a random augmentation set T, the model is trained to learn parameters (θ,ϕ,ρ) such that the predicted assignments ${\left\{{\hat{y}}_{i}\right\}}_{i=1}^{N}$ align with the latent semantic labels ${\left\{y_{i}\right\}}_{i=1}^{N}$ up to a permutation of cluster indices.

## 4. Proposed Method

**Figure 1:**
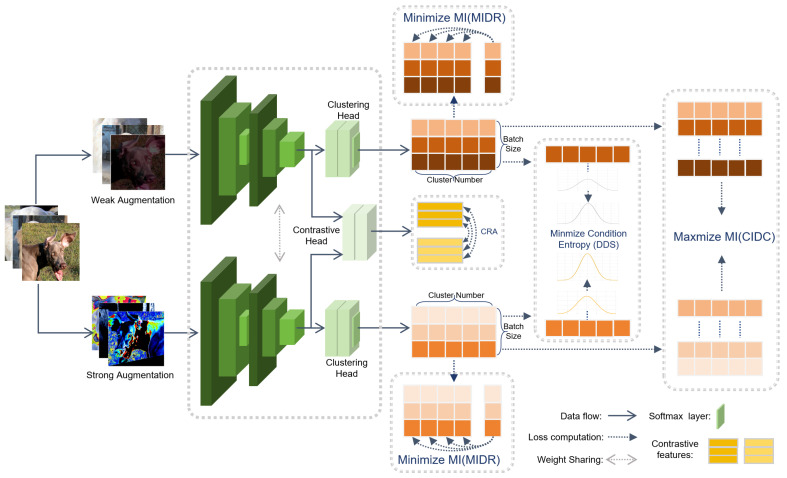
The framework of the proposed ICIRD. The model receives dual-view augmented data, which are processed by a shared encoder to extract representations. These representations are then fed into the clustering head and contrastive head, respectively. In the clustering branch, the DDS, MIDR, and CIDC modules jointly constrain the discriminability, consistency, and redundancy of the predicted distributions. In the contrastive branch, the CRA module aligns cross-view representations at the instance level. The four losses are jointly optimized to obtain discriminative and distributionally balanced clustering results.

The ICIRD framework is designed to maximize discriminative information under a constrained channel, preserving cluster-relevant content while compressing redundant inputs. The DDS module sharpens conditional cluster distributions to produce confident predictions under diverse augmentations, thereby reducing decision uncertainty. The MIDR module minimizes inter-dimensional redundancy within the probability space to decouple categories and improve separability. The CIDC module enforces consistent predictions for identical instances across augmentations, preventing semantic drift and structural fragmentation. The CRA backbone strengthens local semantics and instance discrimination, providing a stable foundation for distribution-level optimization. Together, these four modules form an information-preserving and redundancy-suppressing loop that enables ICIRD to learn sharp, independent, and consistent cluster probabilities, which are directly used as the final clustering outputs (see Figure 1).

### 4.1. Methodological Origins and Contributions

To improve the transparency of methodological origins and to clearly distinguish inherited components from novel contributions, we provide a unified explanation of the four modules that compose the ICIRD framework, namely CRA, CIDC, DDS, and MIDR. While some modules are directly based on established methods, others introduce structural modifications or new formulations tailored for deep clustering. Together, these components enable a principled, information-theoretic optimization of cluster probability distributions.

The CRA module follows the standard formulation of contrastive learning methods such as SimCLR [12] and InfoNCE [10]. It is adopted without modification in this work. Its primary purpose is to provide stable, invariant, and information-bottleneck–oriented feature representations, thereby supplying reliable inputs for subsequent cluster distribution learning rather than introducing a new contrastive objective.

The CIDC module draws directly from the cross-view mutual information maximization principle used in Invariant Information Clustering (IIC) [9]. By enforcing consistent cluster assignment distributions across augmented views of the same instance, CIDC ensures semantic stability under data augmentation. Its form remains consistent with the original IIC formulation, serving as a complementary constraint alongside the discriminative and redundancy-oriented components of ICIRD.

Building upon IMSAT’s discriminative regularization [23], the DDS module introduces a key structural modification. Whereas IMSAT minimizes conditional entropy while maximizing marginal entropy, ICIRD removes the marginal entropy term and retains only conditional entropy minimization. This adjustment is motivated by the fact that CIDC and MIDR already enforce distributional structures related to marginal entropy, making its explicit inclusion redundant. By focusing solely on conditional entropy, DDS provides a clearer and more direct sharpening signal for cluster probability distributions, better aligning with the objectives of deep clustering. This modification constitutes a structural contribution specific to the clustering scenario.

Extending beyond the principles of IIC and CIDC, the MIDR module reconstructs an inter-cluster redundancy measure based on statistical mutual information and minimizes it to suppress redundant dependencies between different cluster assignments. Unlike IIC, which primarily emphasizes cross-view invariance, MIDR explicitly addresses the separability and independence of clusters by reducing inter-cluster statistical overlap. This formulation has not been previously established in deep clustering and therefore represents a novel redundancy-oriented information constraint.

Overall, the main contribution of ICIRD lies not in proposing four entirely new modules, but in formulating a unified, information-theoretic optimization framework for cluster probability distributions. DDS enhances distribution discriminability, MIDR suppresses inter-cluster redundancy, CIDC enforces cross-view invariance, and CRA provides stable representation support. These components collectively yield a structured and complementary optimization of both feature representations and cluster distributions, forming the core innovation of the ICIRD framework.

### 4.2. Discriminative Distribution Sharpness Module

The discriminative mutual information satisfies Equation (3). In fully unsupervised clustering, directly controlling the marginal entropy H(Y) on mini-batches often incurs distributional bias and training instability [40]. In contrast, ICIRD adopts the strategy of minimizing the conditional entropy H(Y∣X) offers a more stable and direct route: it encourages sharper and more confident conditional predictions, thereby—under a controlled H(Y)—equivalently increasing I(X;Y) via its deterministic component [23,39].

For random variables *X* and *Y*, the conditional entropy is:

(4) $$H\left(Y|X\right)=-\sum_{x\in X}p\left(x\right)\sum_{y\in Y}p\left(y|x\right)\log p\left(y|x\right).$$

Since the true P(X) is unknown, it is approximated by the empirical distribution $\hat{p}\left(X\right)$ at the mini-batch or dataset level. With a parameterized encoder and clustering head producing discriminative cluster probabilities $p_{\theta ,\phi }\left(y|x\right)$, and batch size *B*, a Monte-Carlo estimator the conditional entropy:

(5) $$\hat{H}\left(Y|X\right)=-\frac{1}{B}\sum_{i=1}^{B}\sum_{k=1}^{K}p_{\theta ,\phi }\left(y_{k}|x_{i}\right)\log p_{\theta ,\phi }\left(y_{k}|x_{i}\right).$$

Under two augmented views a,b, the variance is reduced by averaging the two conditional entropies and defining the discriminative distribution sharpness (DDS) loss as:

(6) $$L_{\mathrm{DDS}}=\frac{1}{2}\left[\hat{H}\left(Y^{a}\;|X^{a}\right)+\hat{H}\left(Y^{b}\;|X^{b}\right)\right].$$

This loss penalizes flat predictive distributions and compresses conditional uncertainty. Meanwhile, the marginal entropy term H(Y) in the mutual information formulation is handled through the following mechanisms: (i) Multi-view Inter-cluster Distribution Redundancy Reduction (MIDR) module suppresses inter-cluster statistical coupling and effectively increases the usable marginal entropy H(Y), thus forming—together with DDS—a complete, structured maximization of I(X;Y). (ii) Cross-view Instance Distribution Consistency (CIDC) module stabilizes $p_{\theta ,\phi }\left(y|x\right)$ against view perturbations. Combined, these modules mitigate the degeneration and collapse risks inherent to pure entropy minimization and shape sharp, separable, and augmentation-consistent cluster predictions under minimal assumptions.

### 4.3. Multi-View Inter-Cluster Distribution Redundancy Reduction Module

Inspired by IIC [9] and data augmentation, a mutual information-based multi-view inter-cluster distribution redundancy reduction (MIDR) constraint is proposed to minimize mutual information between cluster distributions. This method directly acts on the marginal distributions over clusters, reducing redundant dependencies among cluster probabilities from a probabilistic perspective, thus enhancing the separability and independence of predictive distributions. Compared with conventional deep clustering algorithms that impose constraints on the representation space, MIDR optimizes the cluster assignment layer directly, achieving more effective performance improvement. Moreover, data augmentation helps mitigate the marginal bias introduced by mini-batch sampling, providing stable support for improved discrimination and generalization.

As previously described, a neural network produces a probability matrix $P_{\theta ,\phi }\left(Y|X\right)\in R^{B\times K}$ (abbreviated as *P*), where *B* denotes the batch size and *K* the number of categories. In clustering, the goal is to make the dimensions of this matrix (i.e., its column vectors) as independent as possible to ensure discriminative cluster probabilities. Therefore, the mutual information between category dimensions is minimized to suppress redundancy:

(7) $$min_{i\neq j}I\left(u_{i},u_{j}\right),$$

where $u_{i}$ and $u_{j}$ are two distinct probability columns of *P*. According to the KL-divergence definition of mutual information,

(8) $$I\left(X;Y\right)=E_{p(x,y)}\;\left[\log\frac{p(x,y)}{p(x)p(y)}\right],$$

the joint distribution $P(u_{i},u_{j})$ and the marginal distributions $P(u_{i})$ and $P(u_{j})$ are required. Furthermore, since the correlation between dimensions is positively related to their joint distribution, the joint distribution through inter-dimensional correlations is approximated. The correlation matrix *D* is obtained after column-wise normalization of *P* as $D={\left(P\left(Y|X\right)\right)}^{⊤}P\left(Y|X\right)$. The matrix $D\in R^{K\times K}$ expresses the full inter-dimensional relationships, and once normalized as a probabilistic matrix, it can be used to compute the mutual information.

Data augmentation is then introduced, where $X^{a}$ and $X^{b}$ are two augmented versions of the unlabeled dataset *X*. The parameterized neural network with ϕ and θ produces corresponding probability matrices $P^{a}$ and $P^{b}$. Each row vector in $P^{a}$ or $P^{b}$ represents a sample’s conditional probability over *K* categories, while each column vector estimates the marginal distribution over clusters.

Based on the previous correlation formulation, $D^{a}$ and $D^{b}$ are obtained for each view, where $D_{ij}^{a}$ and $D_{ij}^{b}$ denote the redundancy between dimensions *i* and *j*. Let $\mathrm{sum}(D^{a})$ and $\mathrm{sum}(D^{b})$ denote the total sums of each matrix; they are normalized as $D^{a}=\frac{D^{a}}{\mathrm{sum}(D^{a})}$ and $D^{b}=\frac{D^{b}}{\mathrm{sum}(D^{b})}$. For any matrix *D*, the sum of row *i* gives the marginal $P\left(u_{i}\right)=\sum_{j=1}^{K}D_{ij}$, the sum of column *j* gives $P\left(u_{j}\right)=\sum_{i=1}^{K}D_{ij}$, and the element $D_{ij}$ represents the joint $P(u_{i},u_{j})$. According to the KL-based definition in Equation (8), the inter-cluster distribution redundancy reduction loss (IDR) is given by:

(9) $$L_{\mathrm{IDR}}=\frac{2}{K(K-1)}\sum_{i<j}P\left(u_{i},u_{j}\right)\;\log\;\frac{P(u_{i},u_{j})}{P\left(u_{i}\right)\;P\left(u_{j}\right)}.$$

Because *D* is symmetric, only the upper triangular part is computed to avoid self-correlation and redundancy. In practice, unstable marginals may lead to trivial solutions, so smoothing terms can be added to stabilize training. Extending this formulation to the multi-view case, the MIDR loss becomes:

(10) $$L_{\mathrm{MIDR}}=L_{\mathrm{IDR}}^{a}+L_{\mathrm{IDR}}^{b}.$$

Redundancy reduction is applied only within each view rather than across views, since augmented views may suffer from label permutation inconsistency. In the early stages of training, the cluster assignments of different views for the same instance are still likely to be inconsistent. Although cross-view consistency losses such as CIDC can partially mitigate this issue, enforcing cross-view independence early in training leads to instability before the label spaces align. We will discuss this issue in detail in Section 5.6. By minimizing inter-cluster mutual information in a multi-view setting, the MIDR loss effectively suppresses inter-class redundancy while maintaining semantic consistency, achieving joint optimization of class independence and view consistency. Compared with the single-view IDR, MIDR realizes higher robustness and output stability under strong augmentations.

### 4.4. Cross-View Instance Distribution Consistency Module

A mutual information-based cross-view instance distribution consistency (CIDC) constraint is introduced that is directly applied to the discriminative cluster probabilities, aiming to achieve an unbiased estimation for clustering tasks.

For a data set *X* and its two augmented versions $X^{a}$ and $X^{b}$, after being mapped by parameterized neural networks ϕ and θ, the corresponding discriminative cluster probability matrices are $P(Y^{a}|X^{a})$ and $P(Y^{b}|X^{b})$. We focus on their row vectors $q_{i}^{a}$ and $q_{i}^{b}$ (i∈N), each representing the conditional probability distribution of sample *i* over *K* categories under the respective views. According to the consistency constraint, the cluster probabilities $q_{i}^{a}$ and $q_{i}^{b}$ of the same instance across different views should be similar, which in information theory corresponds to having a large mutual information. Therefore, the mutual information objective between these two vectors can be defined as:

(11) $$\max I(q_{i}^{a},q_{i}^{b}).$$

The joint probability matrix between the two views can be computed as $q_{i}^{a}\cdot {\left(q_{i}^{b}\right)}^{⊤}$. Consequently, the averaged joint probability over all paired instances is expressed as:   

(12) $$Q=\frac{1}{B}\sum_{i=1}^{B}q_{i}^{a}\cdot {\left(q_{i}^{b}\right)}^{⊤},$$

where $Q\in R^{K\times K}$ denotes the cross-view joint probability distribution matrix. Each element $Q_{ij}$ represents the joint probability between category *i* in one view and category *j* in the other. The sum of the *i*-th row corresponds to the marginal probability $Q_{i}$, and the sum of the *j*-th column corresponds to $Q_{j}$. Considering the symmetry of mutual information, i.e., $I\left(q_{i}^{a},q_{i}^{b}\right)=I\left(q_{i}^{b},q_{i}^{a}\right)$, a symmetrized form is adopted in practice: $Q=\frac{1}{2}\left(Q+Q^{⊤}\right)$ Substituting the above formulation into the KL-divergence expression of mutual information, the loss function is given by:

(13) $$L_{CIDC}=-\sum_{i=1}^{K}\sum_{j=1}^{K}Q_{ij}\;\log\frac{Q_{ij}}{Q_{i}Q_{j}}$$

The CIDC loss maximizes the mutual information between cluster probabilities of different augmented views, thereby implicitly enhancing the marginal entropy H(Y) and promoting balanced cluster assignments. By directly aligning semantic distributions at the probabilistic output layer, CIDC achieves a balance between stability and discriminability in clustering and unsupervised classification tasks. Specifically, during the maximization process, according to the mutual information identity $I\left(Y^{a};Y^{b}\right)=H\left(Y^{a}\right)-H\left(Y^{a}|Y^{b}\right)=H\left(Y^{b}\right)-H\left(Y^{b}|Y^{a}\right)$, the marginal entropy is simultaneously maximized. This loss effectively compensates for the deficiency of DDS loss in constraining marginal entropy.

### 4.5. Contrastive Representation Anchoring Module

To enhance the discriminability and robustness of the representation space, a contrastive constraint $L_{\mathrm{CRA}}$ [13,30] is introduced. Given a batch of samples ${\left\{x_{i}\right\}}_{i=1}^{B}$, for each sample, two augmented views are generated $x_{i}^{a},x_{i}^{b}$, where *a* and *b* denote two augmentation policies. In practice, a “weak” augmentation (e.g., random crop, light flip, mild color jitter) and a “strong” augmentation (e.g., heavy color distortion, extreme cropping, large rotation) are used. Through the encoder $f_{\theta }\left(\cdot \right)$ and the contrastive projection head $c_{\rho }\left(\cdot \right)$, the following representations are obtained: $h_{i}^{v}=\mathrm{norm}\left(c_{\rho }\left(f_{\phi }\left(x_{i}^{v}\right)\right)\right),\;v\in \left\{a,b\right\},$ where norm(·) denotes $\ell_{2}$-normalization to stabilize the similarity measure.

Let $\mathrm{sim}\left(u,v\right)=u^{⊤}v$ denote cosine similarity (which coincides with the inner product after $\ell_{2}$ normalization). Let τ>0 be a temperature hyperparameter. For each *i*, the positive sample pair is $\left(h_{i}^{a},h_{i}^{b}\right)$. The negative samples are taken from the other instances in the same batch, ${\left\{h_{j}^{b}\right\}}_{j\neq i}$ (and may be extended via a cross-batch memory bank). The one-direction contrastive objective is:

(14) $$\ell_{i}^{\;a\to b}=-\log\frac{\exp\;\left(\mathrm{sim}(h_{i}^{a},\;h_{i}^{b})/\tau \right)}{\sum_{j=1}^{N}\;\exp\;\left(\mathrm{sim}(h_{i}^{a},\;h_{j}^{b})/\tau \right)}.$$

To avoid directional bias, the objective is symmetrized, and the CRA loss is expressed as:

(15) $$L_{\mathrm{CRA}}=\frac{1}{2N}\sum_{i=1}^{N}\left(\ell_{i}^{\;a\to b}+\ell_{i}^{\;b\to a}\right).$$

CRA provides robust, view-invariant embeddings that reduce noise in discriminative cluster probabilities, improving the reliability of DDS, MIDR, and CIDC estimations. Besides, it indirectly strengthens entropy-based discrimination and cross-view alignment, leading to more stable and separable cluster formation.

### 4.6. Theoretical Interpretation of Objectives

To clarify the theoretical foundation of the information-driven design, this section demonstrates how the three major modules, DDS, MIDR, and CIDC, jointly realize a structured maximization of discriminative mutual information in Equation (3). Specifically, DDS explicitly reduces the conditional entropy, while MIDR and CIDC implicitly increase the marginal entropy, together achieving a balanced optimization of the mutual information components.

The CIDC module maximizes the cross-view mutual information:

(16) $$I\left(Y^{a};Y^{b}\right)=H\left(Y^{a}\right)+H\left(Y^{b}\right)-H\left(Y^{a},Y^{b}\right),$$

where $H\left(Y^{a}\right)\approx H\left(Y^{b}\right)\approx H\left(Y\right)$. Maximizing $I(Y^{a};Y^{b})$ effectively encourages larger H(Y) while suppressing collapse, thereby enhancing balanced cluster utilization and distributional stability across augmented views.

The proposed MIDR objective enhances the marginal entropy H(Y) by reducing inter-cluster redundancy. Let $P_{\theta ,\phi }\left(Y\;|\;X\right)\;\in \;R^{B\times K}$ denote the conditional cluster probability matrix and $\pi_{k}$ the marginal class probability over cluster *k*. When cluster assignments are sharp (near one-hot), the dimensions become mutually exclusive. In this limit, minimizing inter-cluster mutual information drives the marginal distribution π toward uniformity, where H(Y) reaches its maximum. Hence, under sharp assignments, MIDR is equivalent to maximizing H(Y).

In the general soft-assignment case, each column of $P_{\theta ,\phi }\left(Y\;|\;X\right)$ represents a probabilistic cluster dimension, and correlations among these columns imply statistical redundancy between cluster assignments. Minimizing such inter-cluster correlations through MIDR reduces over-dependence among dimensions, encouraging a more uniform marginal distribution $\pi ={\left\{\pi_{k}\right\}}_{k=1}^{K}$, where $\pi_{k}=\sum_{j}{\tilde{D}}_{kj}$. As the distribution flattens, the quadratic term $\sum_{k}\pi_{k}^{2}$ decreases, leading to an increase in the Rényi-2 entropy:

$$H_{2}\left(\pi \right)=-\log\;\sum_{k=1}^{K}\pi_{k}^{2},$$

which provides a lower bound on the Shannon entropy H(Y). Therefore, by decorrelating cluster dimensions and balancing marginal probabilities, MIDR effectively increases (or lower-bounds) H(Y), yielding more independent and evenly distributed cluster assignments.

From the Information Bottleneck (IB) perspective in Equations (1) and (2), the three modules jointly realize the trade-off between *information sufficiency* and *compression*. MIDR and CIDC jointly realize the compression aspect of the IB principle by eliminating nuisance factors in clustering. Specifically, MIDR minimizes inter-dimensional correlations within the cluster probability space, thereby reducing statistical redundancy and encouraging more independent and balanced cluster representations. CIDC, on the other hand, maximizes cross-view invariance to minimize the dependence of cluster assignments on the augmentation variable *T*, effectively filtering out augmentation-induced noise and enforcing semantic consistency across views. Within this framework, DDS plays a complementary role: by minimizing the conditional entropy H(Y∣X), it sharpens predictions and enhances discriminative sufficiency, but does not directly contribute to the compression of I(X;Y).

### 4.7. Information-Principled Objective Formulation

Without loss of generality, the overall objective loss is formulated as follows:

(17) $$L=L_{\mathrm{CRA}}+\lambda_{1}L_{\mathrm{MIDR}}+\lambda_{2}L_{\mathrm{CIDC}}+\lambda_{3}L_{\mathrm{DDS}}$$

where $L_{\mathrm{CRA}}$ is the contrastive representation anchoring loss with Equation (15), $L_{\mathrm{MIDR}}$ is the multi-view inter-cluster distribution redundancy reduction loss with Equation (10), $L_{\mathrm{CIDC}}$ is the cross-view instance distribution consistency loss with Equation (13) and $L_{\mathrm{DDS}}$ is the discriminative distribution sharpness loss with Equation (6). $\lambda_{1},\lambda_{2}$ and $\lambda_{3}$ are the balance hyper-parameters of each loss.

From a theoretical standpoint, the composite objective of ICIRD establishes a cooperative optimization mechanism that integrates representation compactness, distribution independence, and cross-view invariance within a unified information-principled framework. This design maximizes discriminative mutual information between inputs and predictions while suppressing redundant dependencies, thereby yielding compact yet expressive clustering representations. The overall training process is summarized in Algorithm 1.
**Algorithm 1:** ICIRD    **Input**  : Dataset *X*; training epochs *E*; batch size *B*; temperature τ; cluster number *K*; hyper-parameters $\lambda_{1},\lambda_{2},\lambda_{3}$; strong augmentation T, weak augmentation *T*, neural network $g_{\phi },f_{\theta },c_{\rho }$    **Output**: Clustering result ${\left\{\hat{y}\right\}}_{i=1}^{N}$
1**for** epoch=1 **to** *E* **do**2      sample a batch ${\left\{x_{i}\right\}}_{i=1}^{b}$ from *X*;3      obtain two augmentations $X^{a},X^{b}$ according to $X^{a}=T\left(X\right)$ and $X^{b}=T\left(X\right)$;4      compute the representations $Z^{a}$ and $Z^{b}$ by $Z^{a}=f_{\theta }\;\left(X^{a}\right),\;Z^{b}=f_{\theta }\;\left(X^{b}\right)$;5      compute the discriminative probabilities $P^{a},P^{b}$ through clustering head $P^{a}=g_{\phi }\left(Z^{a}\right)$ and $P^{b}=g_{\phi }\left(Z^{b}\right)$;6      compute the contrastive outputs $H^{a},H^{b}$ through contrastive head $H^{a}=c_{\rho }\left(Z^{a}\right)$ and $H^{b}=c_{\rho }\left(Z^{b}\right)$;7      compute DDS loss $L_{\mathrm{DDS}}$ through Equation (6);8      compute MIDR loss $L_{\mathrm{MIDR}}$ through Equation (10);9      compute CIDC loss $L_{\mathrm{CIDC}}$ through Equation (13);10    compute CRA loss $L_{\mathrm{CRA}}$ through Equation (15);11    update ϕ,θ,ρ through gradient descent to minimize in Equation (17);12**end**13Feed the samples ${\left\{x_{i}\right\}}_{i=1}^{N}$ into the network $g_{\phi },f_{\theta }$ to obtain the soft assignments $p_{\theta ,\phi }\left(y_{k}|x_{i}\right)$;14Obtain the labels according to argmax: ${\hat{y}}_{i}=\arg\max_{k}p_{\theta ,\phi }\left(y_{k}|x_{i}\right)$;15**return** ${\left\{\hat{y}\right\}}_{i=1}^{N}$;

## 5. Experiments

### 5.1. Experiment Setting

#### 5.1.1. Datasets

Five well-known benchmark datasets are employed to comprehensively evaluate the effectiveness of the proposed method. CIFAR-10 [41] contains 10 balanced categories of natural images. CIFAR-100 [41] extends CIFAR-10 to 100 fine-grained classes grouped into 20 superclasses, providing a more challenging test of representational capacity and generalization. STL-10 [42] includes 10 categories with higher-resolution images. ImageNet-10 [43] is a subset of the ImageNet dataset comprising 10 semantically distinct categories. ImageNet-Dogs [43] consists of 15 dog breeds and serves as a fine-grained benchmark. Table 1 provides detailed information about these datasets.

#### 5.1.2. Evaluation Metrics

Three widely used metrics are adopted to evaluate the clustering results, including Normalized Mutual Information (NMI) [44], Clustering Accuracy (ACC) [6], and Adjusted Rand Index (ARI) [45]. Note that higher values of the three evaluation metrics indicate better clustering performances.

#### 5.1.3. Implementation Details

Unless otherwise specified, the neural network backbone is based on ResNet-34, which is randomly initialized before training to ensure a fair comparison with other deep clustering algorithms. The original 7 × 7 stride-2 convolution of ResNet is replaced with a 3 × 3 stride-1 convolution, and the max-pooling layer is removed to avoid early downsampling. This adjustment preserves fine-grained spatial information and makes the network better suited for small-resolution datasets such as CIFAR. For ImageNet-10 and ImageNet-Dogs, we resize the images to 224 × 224, while for Stanford-Dogs, we resize them to 96 × 96. Other datasets use their original image resolutions. To accommodate datasets with different image resolutions, the input layer of the model is appropriately modified. The feature dimension of the backbone’s penultimate layer is set to 512 to preserve sufficient representational information. A two-layer MLP with ReLU activation functions is employed as the projection head for contrastive representation learning, producing a unified output dimension of 128. The temperature parameter τ in the contrastive loss is set to 0.5. Another two-layer MLP with Softmax activation is used as the clustering head, whose output dimension corresponds to the number of classes in each dataset, as summarized in Table 1. The model is optimized using the Adam optimizer with a learning rate of $2\times 10^{-4}$ and a weight decay of $1\times 10^{-4}$. The batch size is set to 256, and training is performed for 1500 epochs to ensure convergence. The balance parameters are set to $\lambda_{1}=0.5$, $\lambda_{2}=1$, and $\lambda_{3}=0.5$. Except the analysis of representation dimension conducted on an A100, all experiments are conducted on a workstation equipped with an NVIDIA RTX 3090Ti GPU, a 12th Gen Intel Core i9 CPU, and 64 GB RAM. For data augmentation, the weak augmentation strategy follows SimCLR [12], including RandomResizedCrop, ColorJitter, Grayscale, HorizontalFlip, and GaussianBlur. The strong augmentation strategy follows Strongly Augmented Contrastive Clustering [46], including AutoContrast, Brightness, Color, Contrast, Equalize, Identity, Posterize, Rotate, Sharpness, ShearX/Y, Solarize, and TranslateX/Y.

#### 5.1.4. Comparison Methods

ICIRD was compared with other competing clustering methods, including: VAE [47], JULE [7], DCGAN [48], DEC [6], DAC [18], ID [49], DCCM [24], PICA [50], DRC [51], IDFD [52], CC [13], DCDC [53], DCSC [54], SACC [35], DeepCluE [15], IcicleGCN [36], DCHL [16], CoHiClust [37], CCGCC [17]. Table 2 illustrates the clustering metric results on five image data sets, where the highest and second-highest values are tagged in red and blue, respectively.

### 5.2. Quantitative Analysis of Clustering Results

To evaluate the clustering quality of ICIRD, its performance is presented in comparison with several baseline methods on five commonly used benchmark datasets in Table 2. As shown in the results, ICIRD consistently outperforms most existing methods across all datasets, with particularly outstanding results on CIFAR-100 and ImageNet-Dogs. Both datasets contain a large number of classes with subtle inter-class differences, indicating that ICIRD possesses stronger fine-grained discriminative ability and a superior capacity for modeling complex data distributions. Moreover, ICIRD achieves either the best or second-best performance on the remaining datasets, demonstrating its strong generalization capability and ability to perform well on both coarse-grained and fine-grained clustering tasks.

### 5.3. Clustering on Fine-Grained Dataset

To further verify its applicability in more challenging fine-grained scenarios, additional experiments were conducted on the Stanford-Dogs dataset. Stanford-Dogs [55]: Derived from ImageNet, this dataset contains 120 dog breeds with balanced samples and 96 × 96 images. It features complex backgrounds and fine-grained inter-class variations, posing a challenging benchmark for fine-grained recognition and representation learning.

Several competitive algorithms from Table 2 were selected as comparison methods and reproduced following their publicly recommended configurations. The results, reported in Table 3, clearly show that ICIRD achieves the best performance on this dataset, further confirming its superior discriminability and robustness under highly similar and complex data distributions. This superior performance can be attributed to the redundancy suppression mechanism and discriminative constraint introduced in the clustering head, which jointly promote feature independence in the representation space. In addition, by emphasizing consistency constraints during both representation learning and clustering, ICIRD effectively encourages intra-class compactness, thereby enhancing the separability and stability of the overall clustering structure.

### 5.4. Qualitative Analysis of Clustering Results

#### 5.4.1. Confusion Matrices

**Figure 2:**
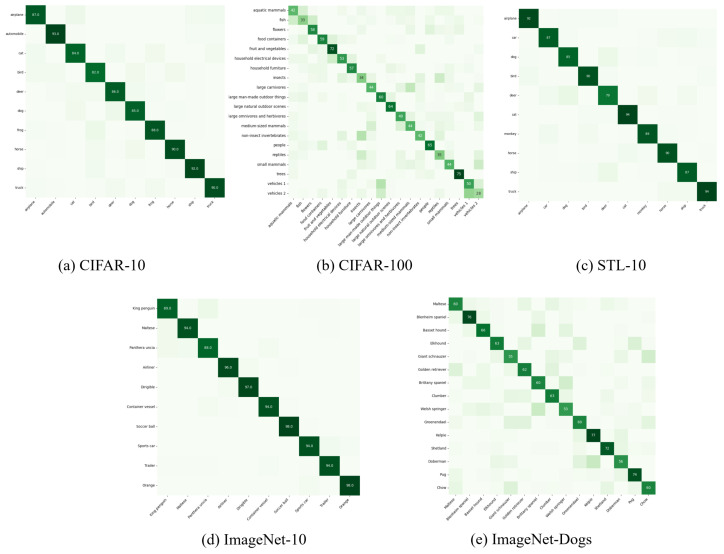
The confusion matrices of ICIRD on five datasets, where the x-axes are the ground-truth labels and the y-axes are the predicted labels. The clearer the diagonal structure in the confusion matrix, the better it is represented.

To further evaluate the class-wise discriminative capability of the proposed model, the confusion matrices on five benchmark datasets are visualized based on the ACC values in Table 2, as shown in Figure 2. These matrices provide an intuitive understanding of the intra-class consistency and inter-class separability achieved by our method across different levels of granularity and visual complexity. Overall, the confusion matrices collectively demonstrate that our model maintains strong discriminative ability under coarse-grained settings and exhibits reasonable generalization to fine-grained categories, although subtle appearance variations among closely related classes remain challenging. This analysis validates the robustness and generality of our clustering framework across diverse visual domains.

#### 5.4.2. Case Studies

**Figure 3:**
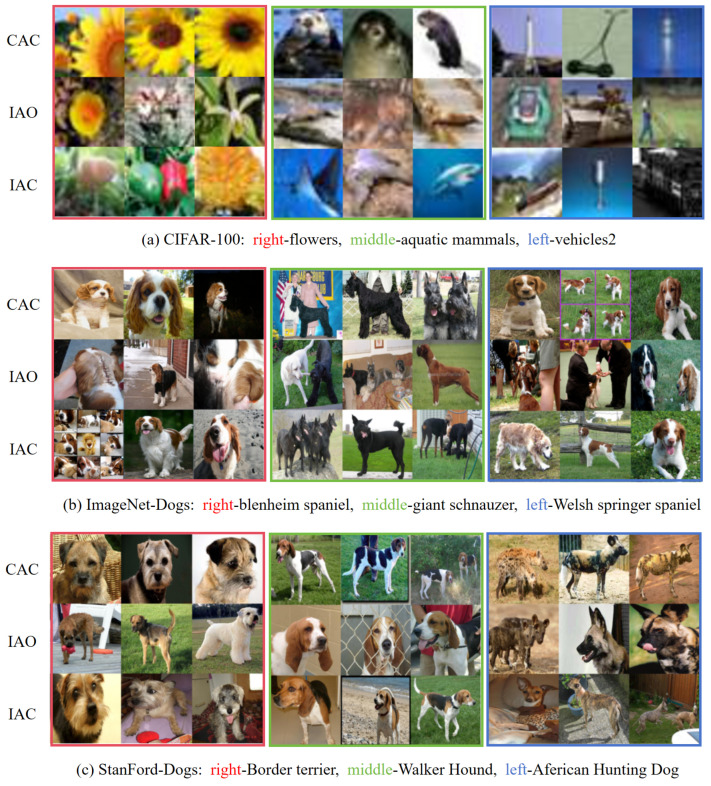
The case studies of ICIRD on CIFAR-100, ImageNet-Dogs and Stanford-Dogs datasets. For each dataset, three classes are visualized, and their samples are highlighted with red, green, and blue bounding boxes corresponding to the left, middle, and right groups in each subfigure. Each row then shows a different case criterion (CAC, IAO, IAC), as indicated on the left side of the subfigure.

In this section, several representative cases from the clustering results are analyzed as illustrated in Figure 3. Specifically, the cases are divided into three categories: (i) cases correctly assigned to their corresponding categories (CAC); (ii) cases incorrectly assigned to other categories (IAO); and (iii) cases incorrectly assigned to the current category (IAC).

Taking the Stanford-Dogs dataset as an example, the model performs well on high-accuracy categories such as the African hunting dog, where distinctive coat patterns and ear shapes clearly differentiate them from other breeds. However, under uncommon viewing angles or in juvenile images, these samples are often misclassified into visually similar categories such as Whippet or Greyhound. For low-accuracy categories like Border Terrier, the model frequently confuses them with breeds such as Norfolk Terrier, Cairn Terrier, and Miniature Schnauzer, all sharing similar facial textures and drooping ears. In the CIFAR-100 dataset, the limited image resolution causes the model to rely primarily on color, texture, and background cues. Consequently, even well-recognized categories such as Flowers are sometimes confused with Fruits and Vegetables or Trees due to color overlap, whereas objects with clean backgrounds and distinct silhouettes tend to be classified more accurately. For the ImageNet-Dogs dataset, misclassifications mainly occur in images containing multiple objects, occlusions, or ambiguous perspectives—such as rear views or extreme close-ups—where discriminative features become less perceptible.

Overall, ICIRD can accurately distinguish subtle differences across fine-grained datasets, while its misclassifications are mainly concentrated in visually ambiguous or difficult cases.

### 5.5. Ablation Studies

#### 5.5.1. Effectiveness Analysis

**Figure 4:**
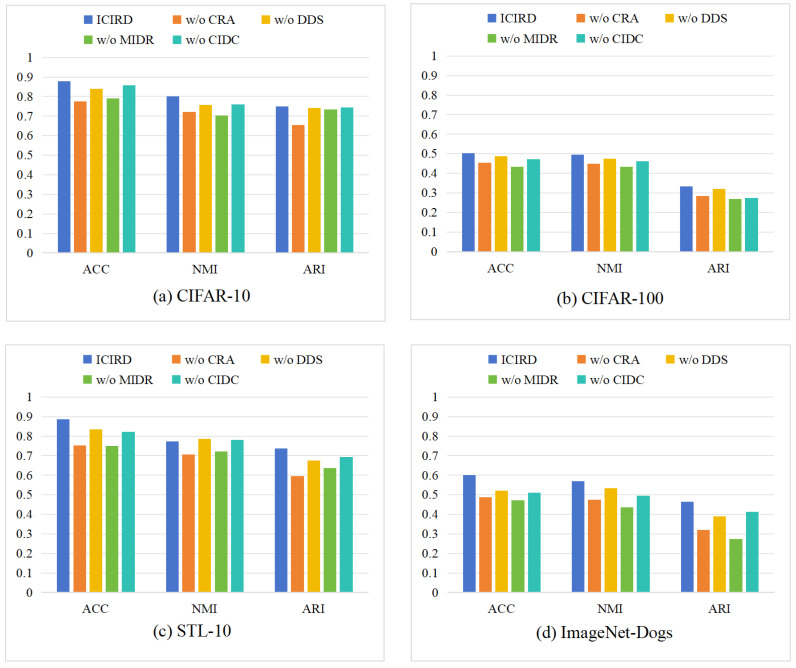
Effectiveness ablation results of the loss functions on four datasets with ACC, NMI, ARI.

In this section, the proposed framework’s losses are evaluated through ablation experiments. Specifically, the following ablation settings are designed to clarify the contribution of each constraint: (i) without the CRA loss; (ii) without the DDS loss; (iii) without the MIDR loss; (iv) without the CIDC loss; and (v) the complete ICIRD model. To comprehensively validate the effectiveness of each constraint, experiments are conducted on CIFAR-10, CIFAR-100, STL-10, and ImageNet-Dogs, which together cover datasets of varying resolution, class number, and granularity. The results are shown in Figure 4. The vertical axis of all subplots is fixed to the range [0, 1] for consistent cross-dataset comparison. It can be observed that removing any loss function leads to a degradation of performance across all datasets, confirming the overall effectiveness of our framework. The importance of each loss is analyzed individually as follows:

(i) When the CRA loss is removed, the performance significantly drops on all datasets, though the model remains stable. This indicates that basic representation learning is necessary for stability, while the remaining three losses still preserve partial discriminative capacity. (ii) When the DDS loss is removed, the decrease is relatively small, implying that it acts as an auxiliary regularization. This aligns with its role in providing stable cluster probabilities for MIDR and CIDC. Even without DDS, the CRA loss can still transfer stability from representation learning to the clustering head. (iii) When the MIDR loss is removed, performance drops substantially across all datasets, showing that this loss has the greatest impact on clustering quality. In this case, only CIDC and DDS impose weak clustering guidance, which is insufficient to maintain strong discriminative structure, yet they still preserve stable performance. (iv) When the CIDC loss is removed, the decline is minor, likely due to partial functional overlap between CIDC and CRA.

Cross-dataset variations are then analyzed to further interpret the ablation results: (i) For the two major losses, CRA and MIDR, we observe that in Figure 4a,c, “w/o CRA” performs worse than “w/o MIDR,” while in Figure 4b,d, the opposite holds. This suggests that MIDR plays a more significant role in fine-grained datasets. (ii) For the two auxiliary losses, DDS and CIDC, Figure 4b,d, show that “w/o DDS” outperforms “w/o CIDC,” whereas Figure 4a shows the reverse. This indicates that CIDC benefits fine-grained datasets, whereas DDS is more effective for low-resolution images.

Overall, all losses in the ICIRD framework contribute positively to the final performance. The four modules complement one another, ensuring robust clustering across datasets with different resolutions and granularity levels.

#### 5.5.2. Convergence Analysis

**Figure 5:**
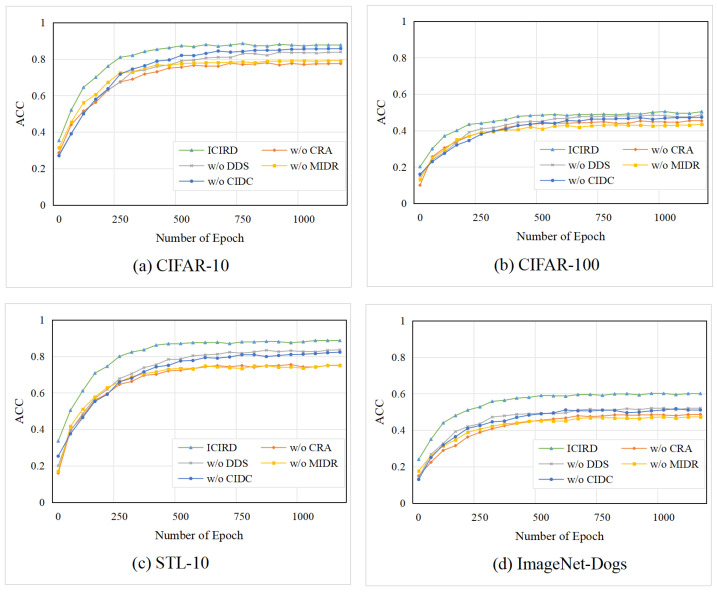
Convergence ablation results of the loss functions on four datasets with ACC.

Furthermore, the convergence curves of ACC across four ablation settings on four datasets were investigated. As illustrated in Figure 5, the ACC values were recorded every 50 epochs to construct line charts. The results demonstrate that ICIRD is consistently outperformed by no other setting throughout the entire training process, confirming the effectiveness and stability of the proposed framework. In addition, it was observed that the CIDC and DDS losses significantly accelerate model convergence, and on certain datasets, their impact on convergence speed and stability even surpasses that of the MIDR loss. These findings further indicate that each loss component within the framework is crucial for enhancing the overall performance of the model.

### 5.6. Discussion of Cross-View IDR

In this section, the feasibility of cross-view IDR (CIDR) is explored and evaluated. The cross-view IDR loss is designed based on the discriminative outputs of two views, where $P^{a}$ and $P^{b}$ are multiplied to obtain $D^{ab}$, and $L_{\mathrm{IDR}}^{ab}$ is subsequently computed according to Equation (9). By further incorporating the MIDR loss introduced in Section 4.3, we obtain a unified multi-view and cross-view IDR objective, termed MCIDR, which is formulated as follows:

(18) $$L_{\mathrm{MCIDR}}=L_{\mathrm{IDR}}^{a}+L_{\mathrm{IDR}}^{b}+\frac{1}{2}\left(L_{\mathrm{IDR}}^{ab}+L_{\mathrm{IDR}}^{ba}\right).$$

where the CIDR loss is $\frac{1}{2}\left(L_{\mathrm{IDR}}^{ab}+L_{\mathrm{IDR}}^{ba}\right)$, and the symmetric cross-view terms are numerically averaged. Ideally, the set of negative samples is expected to be expanded for redundancy reduction through the cross-view mechanism, whereby inter-view associations are established and model performance is further enhanced.

However, the MIDR loss was originally designed as an intra-view constraint because aligning cluster labels across different views in deep learning is inherently challenging. Misaligned cluster probabilities are difficult to compare directly, especially during the early training stages. Nevertheless, the following can be reasonably inferred: (i) In the mid-to-late training stages, the cluster probabilities tend to be stabilized, particularly for samples located near cluster centers. (ii) The CIDC loss is implicitly used to promote distributional alignment across views through consistency constraints. (iii) Noise interference is suppressed by the DDS loss through its sharpening effect. Therefore, the adoption of the CIDR loss can be considered feasible under certain conditions.

In summary, two major factors influence CIDR: (i) the timing of its introduction during training and (ii) the confidence of cluster probabilities. The former is intuitive—CIDR should be introduced in the mid-to-late training stage—and can be controlled via the View Consistency (VC) ratio, while the latter is regulated by the confidence threshold ζ. The view consistency ratio is first defined as follows:

(19) $$\mathrm{VC}=\frac{1}{B}\sum_{i=1}^{B}1\left[\arg\max_{k}p_{i}^{a}\left(k\right)=\arg\max_{k}p_{i}^{b}\left(k\right)\right].$$

Here, $p_{i}^{a}$ and $p_{i}^{b}$ denote the predicted probability distributions of sample *i* under views a and b, respectively. This metric represents the proportion of consistent predictions within a batch and reflects the stability of cluster probabilities. The CIDR loss is introduced only when this ratio exceeds the threshold.

Next, the confidence threshold ζ is defined. Taking a single view as an example, for the filtered probability matrix $P^{\prime }\in R^{B^{\prime }\times K},B^{\prime }<B$, each cluster probability distribution $p_{i}$ in $P^{\prime }$ is normalized using a power function: ${\tilde{p}}_{i}=\frac{p_{i}^{\alpha }}{\sum_{j}^{B^{\prime }}p_{j}^{\alpha }}$, to further mitigate adverse effects. Finally, the processed matrices ${\tilde{P}}^{a}$ and ${\tilde{P}}^{b}$ from the two views are used to compute the CIDR loss following the method described in Section 4.3 and Equation (18).

Next, we conduct experiments to validate the proposed CIDR loss and alignment strategy. Specifically, we compare three settings: ICIRD with MIDR (denoted as $ICIRD_{MIDR}$), ICIRD with MCIDR (denoted as $ICIRD_{MCIDR}$), and ICIRD with MCIDR but without the alignment strategy (denoted as $ICIRD_{MCIDR-}$). In the experiments, VC was set to 0.7 and ζ to 0.8 (α to 2), and then conducted on the CIFAR-10, STL-10, and Stanford-Dogs datasets. The clustering results were reported in Table 4. As shown in the table, $ICIRD_{MCIDR-}$ performs significantly worse than $ICIRD_{MCIDR}$, demonstrating that performing cross-view IDR without the alignment strategy adversely affects model performance. On the other hand, only marginal performance improvements were yielded by the inclusion of the CIDR loss in $ICIRD_{MCIDR}$ on the CIFAR-10 and STL-10 datasets. On STL-10, $ICIRD_{MCIDR}$ was observed to perform slightly worse than $ICIRD_{MIDR}$ in terms of NMI and ARI. However, on the Stanford-Dogs dataset, $ICIRD_{MCIDR}$ was found to significantly outperform $ICIRD_{MIDR}$. This indicates that cross-view redundancy reduction can be beneficial under stable cluster probability conditions, and this scheme is beneficial for discrimination on fine-grained datasets. How to obtain more stable cluster probabilities remains worth investigating.

### 5.7. Analysis of Hyper-Parameters

#### 5.7.1. Analysis of Representation Dimension

**Figure 6:**
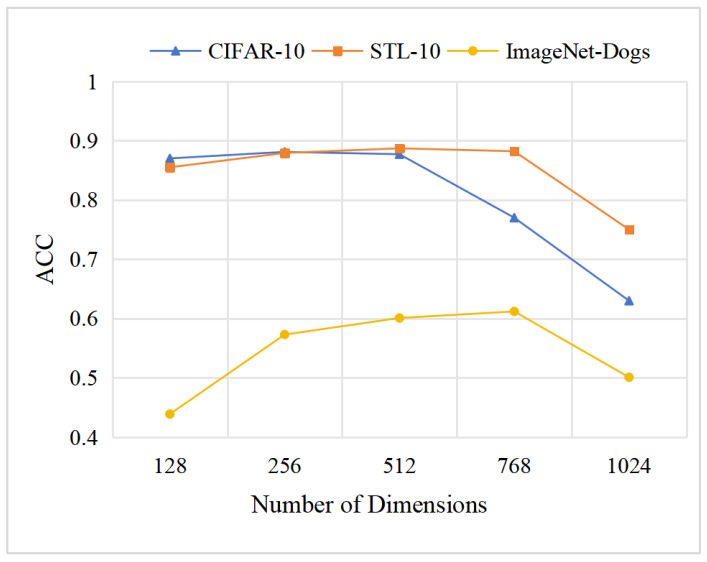
Influence of different representation dimension on three datasets.

The choice of representation dimensionality directly influences the optimization stability and information regularization of each module. To analyze this effect, we evaluate MCIDR under varying representation dimensionalities in {128,256,512,768,1024} across three datasets (CIFAR-10, STL-10, and ImageNet-Dogs), with the results shown in Figure 6. As observed, larger representation dimensions improve MCIDR’s performance on high-resolution or fine-grained datasets. However, excessively large dimensions cause performance degradation. This degradation can be attributed to two factors: (i) Higher dimensionality introduces greater spatial redundancy, making redundancy reduction more difficult. (ii) Excessive dimensionality may exceed the representational capacity of ResNet-34.

#### 5.7.2. Analysis of Cluster Number

**Figure 7:**
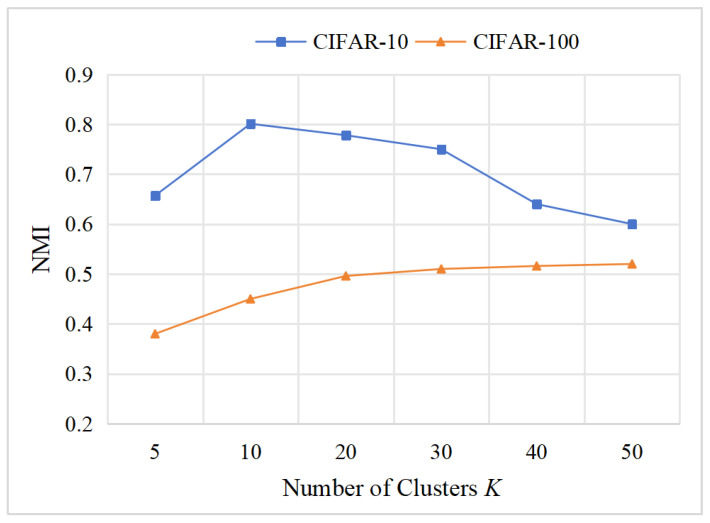
Influence of predefined cluster number on CIFAR-10/100 datasets.

In previous experiments, the number of clusters *K* was predefined. However, in real-world scenarios, such prior knowledge is often unavailable. To evaluate the sensitivity of MCIDR to semantic granularity and the robustness of its distributional constraints, we varied the value of *K* with the value in {5,10,20,30,40,50} on the CIFAR-10 and CIFAR-100 datasets and reported the corresponding NMI results, as shown in Figure 7. The results indicate that when K deviates from the true number of categories in CIFAR-10, the NMI drops sharply, suggesting that MCIDR tends to learn cluster structures consistent with the intrinsic category structure of the data. In contrast, for CIFAR-100, the NMI steadily increases as *K* grows. This aligns with the dataset’s hierarchical organization, where 100 subclasses are grouped under 20 superclasses. This experiment demonstrates that MCIDR effectively captures underlying semantic structures.

#### 5.7.3. Analysis of Balance Parameters

**Figure 8:**
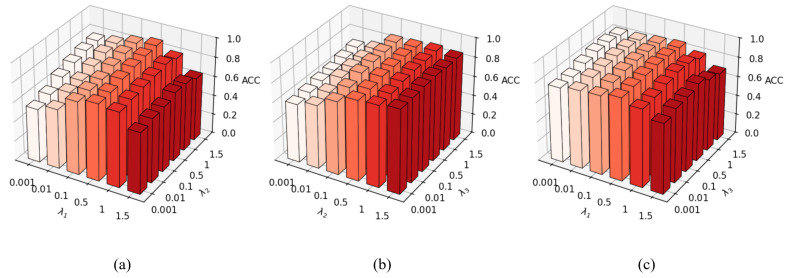
(**a**–**c**) Three-dimensional bar chart analysis of balance parameters $\lambda_{1},\lambda_{2},\lambda_{3}$ on CIFAR-10 datasets.

To further investigate the relationships among the three balancing parameters $\lambda_{1}$, $\lambda_{2}$, and $\lambda_{3}$ in the total loss of MCIDR and their influence on experimental results, we construct the visualization shown in Figure 8 based on the CIFAR-10 dataset. Each parameter is sequentially set to the values in {0.001,0.01,0.1,0.5,1,1.5}. (i) In Figure 8a, an increase in $\lambda_{1}$ gradually improves model performance; however, when $\lambda_{1}>1$, the performance begins to degrade. This may be attributed to unstable cluster probabilities during the early stages of joint training, where an excessively large $\lambda_{1}$ can impair model stability. (ii) In contrast, a higher $\lambda_{2}$ generally enhances model performance except when $\lambda_{1}>1$. This indicates that the CIDC loss positively influences MIDR and effectively improves overall performance. Furthermore, it suggests that the MIDR loss has a more significant impact on the framework than the CIDC loss. (iii) Figure 8b further reveals a clear positive correlation between CIDC and DDS: when one of these parameters remains fixed, model performance increases with the other until convergence plateaus. (iv) According to Figure 8c, the relationship between $\lambda_{1}$ and $\lambda_{3}$ is similar to that between $\lambda_{1}$ and $\lambda_{2}$, suggesting implicit synergy between the two losses. Moreover, the framework is more sensitive to an excessively large $\lambda_{1}$ than to a smaller one. In summary, the loss terms in MCIDR exhibit complementary and mutually reinforcing effects.

## 6. Illustrative Applications and Scalability of ICIRD

In this section, we first discuss the scalability of ICIRD across different data modalities and then discuss its practical application in various downstream tasks.

### 6.1. Extending ICIRD to Non-Image Modalities

In this study, we primarily conduct experiments on image datasets and adopt ResNet as the backbone feature extractor. When extending ICIRD to other modalities such as textual data or structured tabular data, the main challenges arise from the selection of an appropriate backbone architecture and the design of data augmentation (i.e., multi-view construction) strategies. It is worth noting that, aside from the contrastive learning component, which depends on modality-specific augmentations, the other three information-theoretic modules of ICIRD (DDS, MIDR, and CIDC) remain structurally unchanged across different data types. Their sensitivity to modality is minimal, and thus only minor adaptations based on input feature formats and view-construction strategies are required.

For textual datasets, widely used Transformer-based encoders such as BERT [56], DeBERTa-v3 [57], or more recent variants such as the model studied by Guedes and da Silva [58] can be employed. Multi-view textual augmentations are typically constructed through dropout masking, span masking, synonym substitution, or sentence-level perturbation; these augmentation strategies have been demonstrated to be effective in unsupervised text clustering, as shown in SimCSE [59] and CoCLR-text [60].

For structured behavioral or attribute-based tabular data, Transformer architectures specifically designed for tabular modeling, such as TabTransformer [61] and FT-Transformer [62], can be adopted. In unsupervised settings, multi-view construction for tabular data often relies on feature dropout, feature-subset sampling, or noise injection, similar to the masking-based pseudo-view strategy introduced in TabularSSL [63]. ICIRD can operate directly on such backbone–augmentation pipelines and produce stable and consistent cluster distributions across samples.

### 6.2. Practical Application of ICIRD in Downstream Tasks

To clarify how ICIRD functions in practical scenarios, we next discuss its behavior across representative downstream tasks and the adaptations required in each setting. In image recognition tasks, deep clustering methods such as ICIRD primarily contribute in two core directions: unsupervised clustering and novel category discovery. For unsupervised clustering, one typically assumes a reasonable estimate of the true number of categories in order to set the dimensionality of the clustering head. The model is then trained using the standard ICIRD configuration to learn cluster probability distributions, which subsequently serve as pseudo-labels for downstream image recognition. In novel category discovery scenarios, a single supervised warm-up on the labeled subset is first performed—an established practice in prior work, including UNO [64] and GCD [65]. After this warm-up stage, ICIRD’s information-theoretic optimization is applied to the unlabeled subset, enabling the model to construct a stable cluster structure under a fixed number of unknown categories K and to produce reliable predictions for the novel classes.

In other domains, such as medical diagnostics and customer segmentation, ICIRD can be applied in essentially the same manner as in image recognition. The four information-theoretic modules of ICIRD typically require no structural modification. Only “outer-layer” adaptations are needed—such as replacing the backbone, redesigning the view-construction strategy, or adjusting loss weights—to accommodate the characteristics of each modality.

When a task simultaneously requires high-quality feature embeddings and reliable clustering outputs, certain modifications to ICIRD become necessary. One feasible approach is to shift the MIDR component from the cluster-distribution space to the representation space, thereby computing inter-cluster mutual-information redundancy directly on feature embeddings to enhance their linear separability. In addition, the DDS module can be strengthened—for instance, by introducing a marginal-entropy term—to compensate for the absence of MIDR at the probability-distribution level, forming a more complete discriminative mutual-information objective that stabilizes the clustering assignments. Under such a configuration, however, ICIRD effectively transitions into a representation-oriented deep clustering framework, and thus, the entire model requires comprehensive re-evaluation and re-adjustment to ensure consistency and effectiveness.

In scenarios where cross-modal data inevitably arises, ICIRD still does not require structural modifications to its four information-theoretic modules. Instead, the additional modality can simply be incorporated as an extra form of data augmentation and integrated into the multi-view framework. This design is consistent with recent advances in cross-modal contrastive learning, such as the CLIP-style [66] modality alignment used in MAC [67], which provides a practical reference for extending ICIRD to multimodal settings.

## 7. Conclusions

This paper proposes ICIRD, a deep clustering framework that formulates clustering as a process of optimizing the informational structure of cluster probability distributions. ICIRD jointly regularizes feature representations and cluster assignments through information preservation, redundancy reduction, and cross-view consistency, enabling the model to learn sharp, independent, and invariant cluster distributions. The framework establishes a controllable balance between information retention and compression, ensuring stable and bounded optimization. Extensive experiments on multiple benchmarks validate that ICIRD achieves superior clustering accuracy, robustness compared with existing methods. In addition, ICIRD investigates the role of view alignment in improving clustering consistency and robustness across augmented views. Overall, ICIRD provides a unified and principled paradigm for information-theoretic deep clustering, offering both theoretical insight and practical guidance for future research in representation learning and unsupervised semantic modeling.

## Figures and Tables

**Table 1:** The detailed information of datasets used in experiments.

Dataset	Class	Images Number	Image Size
CIFAR-10	10	60,000	32 × 32 × 3
CIFAR-100	20/100	60,000	32 × 32 × 3
STL-10	10	13,000	96 × 96 × 3
ImageNet-10	10	13,000	224 × 224 × 3
ImageNet-Dogs	15	19,500	224 × 224 × 3

**Table 2:** Clustering performance by different competing clustering algorithms on five data sets.

Datasets	CIFAR-10			CIFAR-100			STL-10			ImageNet-10			ImageNet-Dogs		
Metric	ACC	NMI	ARI	ACC	NMI	ARI	ACC	NMI	ARI	ACC	NMI	ARI	ACC	NMI	ARI
VAE [47]	0.291	0.245	0.167	0.152	0.108	0.040	0.282	0.200	0.146	0.334	0.193	0.168	0.179	0.107	0.079
JULE [7]	0.272	0.192	0.138	0.137	0.103	0.033	0.277	0.182	0.164	0.300	0.175	0.138	0.138	0.054	0.028
DCGAN [48]	0.315	0.265	0.176	0.151	0.120	0.045	0.298	0.210	0.139	0.346	0.225	0.157	0.174	0.121	0.078
DEC [6]	0.301	0.257	0.161	0.185	0.136	0.050	0.359	0.276	0.186	0.381	0.282	0.203	0.195	0.122	0.079
DAC [18]	0.522	0.396	0.306	0.238	0.185	0.088	0.470	0.366	0.257	0.527	0.394	0.302	0.275	0.219	0.111
ID [49]	0.440	0.309	0.221	0.267	0.221	0.108	0.514	0.362	0.285	0.632	0.478	0.420	0.365	0.248	0.172
DCCM [24]	0.623	0.496	0.408	0.327	0.285	0.173	0.482	0.376	0.262	0.710	0.608	0.555	0.383	0.321	0.182
PICA [50]	0.696	0.591	0.512	0.337	0.310	0.171	0.713	0.611	0.531	0.870	0.802	0.761	0.352	0.352	0.201
DRC [51]	0.727	0.621	0.547	0.367	0.356	0.208	0.747	0.644	0.569	0.884	0.830	0.798	0.389	0.384	0.233
IDFD [52]	0.815	0.711	0.663	0.425	0.426	0.264	0.756	0.643	0.575	0.954	0.898	0.901	0.591	0.546	0.413
CC [13]	0.790	0.705	0.637	0.429	0.431	0.266	0.850	0.764	0.726	0.893	0.859	0.822	0.429	0.445	0.274
DCDC [53]	0.699	0.585	0.506	0.349	0.310	0.179	0.734	0.621	0.547	0.879	0.817	0.787	0.365	0.360	0.207
DCSC [54]	0.798	0.704	0.644	0.469	0.452	0.293	0.865	**0.792**	**0.749**	0.904	0.867	0.838	0.443	0.462	0.299
SACC [35]	0.851	0.765	0.724	0.443	0.448	0.282	0.759	0.691	0.626	0.905	0.877	0.843	0.437	0.455	0.285
DeepCluE [15]	0.764	0.727	0.646	0.457	0.472	0.288	-	-	-	0.924	0.882	0.856	0.416	0.448	0.273
IcicleGCN [36]	0.807	0.729	0.660	0.461	0.459	0.311	-	-	-	**0.955**	0.904	0.905	0.415	0.456	0.279
DHCL [16]	0.801	0.710	0.654	0.446	0.432	0.275	0.821	0.726	0.680	-	-	-	0.511	0.495	0.359
CoHiClust [37]	0.839	0.779	0.731	0.437	0.467	0.229	0.613	0.584	0.474	0.953	**0.907**	0.899	0.355	0.411	0.232
CCGCC [17]	0.864	0.778	0.742	0.482	0.486	0.316	0.779	0.698	0.645	0.904	0.859	0.833	0.579	0.568	0.449
**ICIRD**	**0.877**	**0.801**	**0.750**	**0.504**	**0.496**	**0.333**	**0.887**	0.774	0.737	0.945	0.893	**0.906**	**0.601**	**0.571**	**0.464**

The highest and second highest values are tagged in **bold** and underline, respectively.

**Table 3:** Clustering performance on Stanford-Dogs dataset.

Dataset	ACC	NMI	ARI
DCSC	0.097	0.259	0.034
IDFD	0.132	0.331	0.062
CCGCC	0.140	0.341	0.059
IcicleGCN	0.082	0.235	0.035
DHCL	0.105	0.316	0.064
**ICIRD**	**0.176**	**0.366**	**0.101**

The highest and second highest values are tagged in **bold** and underline, respectively.

**Table 4:** Performance comparison with $ICIRD_{MIDR}$, $ICIRD_{MCIDR}$, and $ICIRD_{MCIDR-}$ on three datasets.

Datasets	CIFAR-10			STL-10			Stanford-Dogs		
Meric	ACC	NMI	ARI	ACC	NMI	ARI	ACC	NMI	ARI
$ICIRD_{MIDR}$	0.877	0.801	0.750	0.887	**0.774**	**0.737**	0.176	0.366	0.101
$ICIRD_{MCIDR}$	**0.886**	**0.815**	**0.753**	**0.894**	0.765	0.721	**0.194**	**0.391**	**0.109**
$ICIRD_{MCIDR-}$	0.852	0.768	0.725	0.864	0.747	0.708	0.139	0.317	0.083

The highest values are tagged in **bold**.

## Data Availability

The original data presented in the study are openly available in CIFAR-10 (https://www.cs.toronto.edu/~kriz/cifar.html, (accessed on 3 November 2025)), STL-10 (https://cs.stanford.edu/~acoates/stl10/, (accessed on 3 November 2025)), ImageNet (https://image-net.org/download-images.php, (accessed on 3 November 2025)), Stanford-Dogs (http://vision.stanford.edu/aditya86/ImageNetDogs/main.html, (accessed on 3 November 2025)).

## Abbreviations

The following abbreviations are used in this manuscript:

ICIRD	Information-principled Deep Clustering for Invariant, Redundancy-reduced and Discriminative Cluster Distributions
DDS	Discriminative Distribution Sharpness
MIDR	Multiview Inter-cluster Distribution Redundancy Reduction
CIDC	Cross-view Instance Distribution Consistency
CRA	Contrastive Representation Anchoring
MI	Mutual Information
DPI	Data Processing Inequality
IDR	Inter-cluster Distribution Redundancy
KL	Kullback–Leibler Divergence
